# Anti-inflammatory effects of ozenoxacin, a topical quinolone antimicrobial agent

**DOI:** 10.1038/s41429-020-0278-5

**Published:** 2020-01-23

**Authors:** Keisuke Tabara, Rie Tamura, Aki Nakamura, Sachi Mori, Takamichi Kitano, Koki Fujikawa, Mika Fujikawa, Kazuaki Okamoto, Shoji Kanayama, Hideya Uratsuji, Fumiaki Ikeda, Tatsumi Matsumoto

**Affiliations:** Maruho Co., Ltd, Kyoto R&D Center, Drug Development Laboratories, Kyoto Research Park, Bldg. #5, 93 Chudoji Awata-cho, Shimogyo-ku, Kyoto, 600-8815 Japan

**Keywords:** Inflammatory diseases, Inflammation, Pharmacology

## Abstract

Ozenoxacin is a topical quinolone showing potent antimicrobial activities against Gram-negative and Gram-positive bacteria and is widely used for the treatment of inflammatory acne. However, the anti-inflammatory activities of ozenoxacin have not been examined so far. In the present study, we investigated the in vitro and in vivo anti-inflammatory effects of ozenoxacin. The production of interleukin (IL)-6 and IL-8 by human epidermal keratinocytes stimulated by heat-killed *Cutibacterium acnes* was significantly inhibited by ozenoxacin at concentrations from 1 to 30 μg ml^−1^. Likewise, the production of IL-6, IL-8, and tumor necrosis factor alpha by stimulated THP-1 cells, a human monocyte cell line, was inhibited by ozenoxacin at concentrations from 1 to 30 μg ml^−1^. The production of IL-1β by THP-1 was also inhibited by ozenoxacin at the concentration of 30 μg ml^−1^. Phosphorylation of the mitogen**-**activated protein kinases and degradation of IκB-α, an inhibitory factor of NF-κB in keratinocytes and THP-1 cells, was increased by stimulation with heat-killed *C. acnes*. Of these activated intracellular pathways, the p38 phosphorylation pathway was remarkably reduced by ozenoxacin in both keratinocytes and THP-1 cells. In addition, the application of 2% ozenoxacin suppressed the increase in the ear thickness of rats induced by an intracutaneous injection of heat-killed *C. acnes*. These findings suggest that ozenoxacin possesses an anti-inflammatory activity, which may contribute to its therapeutic effects on inflammatory acne.

## Introduction

Acne vulgaris is one of the most common skin diseases in adolescents and young adults, and an estimated up to 80% of the population is affected by the condition at some point in their lives [1]. Acne pathogenesis begins with abnormal keratinization in the hair follicle infundibulum leading to follicular occlusion and noninflammatory comedone formation, and comedones develop into papules and pustules, so-called inflammatory acne. Although many factors, such as increase or change of sebum composition, hormone balance, and change of skin microflora, may be involved in acne pathogenesis [2], the precise pathogenesis of acne remains unclear.

However, it is widely accepted that innate immune response to *Cutibacterium acnes* (formerly known as *Propionibacterium acnes*), a Gram-positive skin commensal bacterium, is involved in inflammatory acne. The mechanisms underlying the *C. acnes-*induced inflammatory response of immune cells, such as keratinocytes, neutrophils, and macrophages, are possibly similar to those underlying the comparable responses induced by other Gram-positive bacteria. Peptidoglycan and lipoteichoic acid, which are predominant in the composition of cell walls of Gram-positive bacteria, stimulate toll-like receptors (TLRs) [3]. The stimulation of TLRs induces activation of mitogen-activated protein kinases (MAPKs), such as extracellular signal-related kinases (ERK1/2), p38 MAPK, and c-Jun N-terminal kinases (JNK), and translocation of NF-κB from the cytoplasm to nucleus, which result in the production of some pro-inflammatory cytokines [4]. In vitro studies showed that *C.* acnes induced the production of pro-inflammatory cytokines, such as interleukin (IL)-1, IL-6, IL-8, and tumor necrosis factor alpha (TNF-α) via TLR activation in human keratinocytes, sebocytes, and monocytes [5–8]. It is well known that these cytokines induce neutrophil migration to pilosebaceous follicles and T-cell activation, which cause more severe inflammation [9, 10]. In addition, macrophages expressing TLR2 on the cell surface surround pilosebaceous follicles, and the expression of pro-inflammatory cytokines is elevated in acne lesions [5, 11–13]. From the above evidence, it is important for treatment of inflammatory acne to not only eradicate *C. acnes* but also reduce inflammation.

Topical antimicrobial agents, such as clindamycin and nadifloxacin, are often used for inflammatory acne treatment [14–16]. It is known that these agents have anti-inflammatory effects. For instance, clindamycin has been shown to suppress the production of IL-1β and interferon-γ (IFN-γ) by peripheral blood mononuclear cells (PBMC) stimulated by *C. acnes* [17]. Nadifloxacin has been shown to suppress the production of pro-inflammatory cytokines by keratinocyte and PBMC [17].

Ozenoxacin, a novel nonfluorinated quinolone, has demonstrated a broad antimicrobial spectrum against both Gram-positive and Gram-negative bacteria, including *C. acnes* [18–23]. Recently, a lotion containing 2% ozenoxacin (Zebiax Lotion 2%, Maruho Co., Ltd, Osaka, Japan) has demonstrated a good therapeutic effect on patients with acne vulgaris in Japan [24]. Therefore, in the Japanese Guideline for the Treatment of Acne Vulgaris 2017, ozenoxacin is strongly recommended for the treatment of inflammatory acne as an external agent, as well as clindamycin and nadifloxacin [14]. However, in contrast to clindamycin, nadifloxacin, and other antimicrobial agents, the anti-inflammatory effects of ozenoxacin have not been studied so far, and the data that explain the mechanism of therapeutic effects of ozenoxacin for inflammatory acne are limited.

In the present study, we used human keratinocyte and monocyte cell lines and a rat model of acute dermatitis to investigate the anti-inflammatory effects of ozenoxacin and evaluate the possible contribution of these effects to anti-inflammatory acne therapy.

## Materials and methods

### Reagents

Humedia-KB2 was purchased from Kurabo Industries Ltd (Osaka, Japan). RPMI-1640, Opti-MEM, fetal bovine serum (FBS), protease/phosphatase inhibitor cocktail, RIPA buffer, and SuperSignal West Dura Extended Duration Substrate were purchased from Thermo Fisher Scientific, Inc. (Waltham, MA, USA). Anaero Columbia agar with rabbit blood was purchased from Nippon Becton Dickinson Company, Ltd (Tokyo, Japan). Cell Count Reagent (WST-1) and blocking solution were purchased from Nacalai Tesque, Inc. (Kyoto, Japan). ELISA kits for human and rat IL-1β, IL-6, IL-8, and TNF-α were purchased from R&D Systems, Inc. (Minneapolis, MN, USA). Anti-human-ERK1/2, phospho-ERK1/2, p38, phospho-p38, phospho-JNK, IκB-α, and GAPDH antibodies were purchased from Cell Signaling Technology, Inc. (Danvers, MA, USA). Anti-human-JNK antibody was purchased from Abcam (Cambridge, UK). Ozenoxacin was obtained from Toyama Chemical Co., Ltd (Tokyo, Japan). Nadifloxacin was purchased from Tokyo Chemical Industry Co., Ltd (Tokyo, Japan). Clindamycin hydrochloride was purchased from Sigma-Aldrich Co., Ltd (St. Louis, MO, USA).

### Preparation of heat-killed *C. acnes*

*C. acnes* strain used in the present study was isolated from Japanese patients with acne vulgaris in 2013. This strain was used in accordance with the Ethical Guidelines for Epidemiological Research (issued on the December 1, 2007, partial revision), and patients’ information was anonymized. This strain was cultured on Anaero Columbia agar with rabbit blood at 35 °C for 3–5 days under anaerobic conditions using the AnaeroPack system (Mitsubishi Gas Chemical Company, Inc., Tokyo, Japan). The bacteria were washed and resuspended in sterile water and heated at 95 °C for 5 min. Heat-killed bacterial suspensions were freeze-dried using a freeze dryer (Tokyo Rikakikai Co., Ltd, Tokyo, Japan) and stored at room temperature until use.

### Cell culture

Human Epidermal Keratinocytes, adult (HEKa cells) (Thermo Fisher Scientific, Inc.) were cultured in Humedia-KB2 supplemented with 10 μg ml^−1^ insulin, 0.1 ng ml^−1^ epidermal growth factor, 0.4% bovine pituitary extract, 0.5 μg ml^−1^ hydrocortisone, 50 μg ml^−1^ gentamicin sulfate, and 50 ng ml^−1^ amphotericin B. The human monocyte cell line THP-1 (ECACC, Wiltshire, UK) cells were cultured in RPMI-1640 medium supplemented with 10% heat-inactivated FBS, 100 U ml^−1^ penicillin, and 100 μg ml^−1^ streptomycin. These cells were cultured at 37 °C in a 5% CO_2_ incubator.

### ELISA for measuring pro-inflammatory cytokine production by HEKa and THP-1 cells

HEKa cells were plated into 24-well plates at the density of 1 × 10^4^ cells/cm^2^ and were cultured at 37 °C in an atmosphere of 5% CO_2_. After 24 h, the medium was replaced by the Humideia-KB2 without supplements and cultured for another 24 h. Cells were cultured with heat-killed *C. acnes* (500 μg ml^−1^: ~1 × 10^9^ CFU ml^−^^1^) and ozenoxacin, nadifloxacin, and clindamycin at concentrations of 1, 3, 10, and 30 μg ml^−1^ for up to 24 h. THP-1 cells were seeded into Opti-MEM in 24-well plates at a density of 2 × 10^5^ cells/cm^2^ and cultured with heat-killed *C. acnes* (50 μg ml^−1^: ~1 × 10^8^ CFU ml^−1^) and antimicrobials at 37 °C in an atmosphere of 5% CO_2_ for up to 24 h. Cytokine levels in culture supernatants were measured by ELISA in accordance with the manufacturer’s instructions. Inhibition rate (%) was calculated using the equation: Inhibition rate = (1−cytokine concentration in the control group/cytokine concentration in the treated group) × 100.

### Cytotoxicity of antimicrobial agents for HEKa and THP-1 cells

HEKa cells and THP-1 cells were cultured under the same condition of the cytokine production assay. After culturing with each antimicrobial agent, cell viability was measured by WST-1 reagent. The measurement was proceeded according to the manufacturer’s instructions.

### Western blot analysis of TLR-related cell signaling

HEKa cells and THP-1 cells were cultured with heat-killed *C. acnes* (500 μg ml^−1^ for HEKa and 50 μg ml^−1^ for THP-1, respectively) and 30 μg ml^-1^ of ozenoxacin or 30 μg ml^-1^ of nadifloxacin at 37 °C in an atmosphere of 5% CO_2_ for 20 or 60 min. The cells were rinsed with ice-cold PBS and lysed in RIPA buffer supplemented with protease/phosphatase inhibitor cocktail, and 10 μg of cell lysate proteins was separated by SDS-PAGE and transferred into PVDF membranes (Bio-Rad Laboratories, Inc., Hercules, CA, USA). The membranes were incubated in blocking solution and incubated with anti-ERK1/2 antibody, anti-phospho-ERK1/2 antibody, anti-p38 antibody, anti-phospho-p38 antibody, anti-JNK antibody, anti-phospho-JNK antibody, anti-IκB-α antibody, and anti-GAPDH antibody at 4 °C overnight. The PVDF membranes were washed in TBST, incubated with horseradish peroxidase-conjugated secondary antibodies at room temperature for 1 h, and the bands were visualized with SuperSignal West Dura Extended Duration Substrate. The signal intensities of bands were measured using Multi Gage Ver3.0 (FUJIFILM Corporation, Tokyo, Japan) and normalized to the GAPDH level.

### Animals

Male Sprague-Dawley rats were purchased from Charles River Laboratories Japan, Inc. (Kanagawa, Japan) and used at 7 weeks old. The animals were allowed free access to water and standard laboratory food and housed at a temperature of 23 ± 3 °C and relative humidity of 50 ± 20%, under a 12 h light/12 h dark cycle. All of the animal experimental procedures were approved by the Ethics Committee for Animal Experiments of Maruho Co., Ltd, and conducted in accordance with the Guiding Principles for the Care and Use of Laboratory Animals at Maruho Co., Ltd.

### Rat model of *C. acnes*-induced acute dermatitis

Heat-killed *C. acnes* (25 μg in 20 μl: ~4 × 10^7^ CFU 25 μg^−1^) or saline alone was intradermally injected into the ventral side of right ear using a microsyringe under isoflurane anesthesia [25, 26]. The 2% ozenoxacin-containing lotion or the lotion base was administered topically to the surface of the right ear of rats just before intradermal injection. The ear thickness was measured using a digital thickness gauge (Mitutoyo Corporation, Kanagawa, Japan) at 2 h after intradermal injection.

### Statistical analysis

The statistical significance of differences between the means of measured parameters for the control and treated group was determined by Dunnett’s multiple comparison test or Student’s or Welch’s *t-*test. Mean differences were considered to be statistically significant when *P* < 0.05. Analyses were performed with the statistics software EXSUS, ver. 8.1 (CAC Croit Corporation, Tokyo, Japan).

## Results

### Effects of ozenoxacin on heat-killed *C. acnes*-induced pro-inflammatory cytokine production in human keratinocytes

The production of IL-6 and IL-8 in HEKa cells was increased after exposure to heat-killed *C. acnes* concentration dependently (from 100 to 1000 μg ml^−1^, data not shown). The effects of antimicrobial agents on pro-inflammatory cytokine production in HEKa cells were evaluated after exposure to 500 μg of the bacteria per ml (~1 × 10^9^ CFU ml^−1^).

**Fig. 1:**
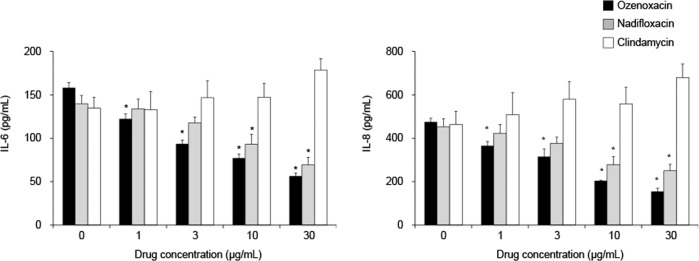
Effects of antimicrobial agents on heat-killed *C*. *acnes*-induced cytokine production in human epidermal keratinocytes, adult (HEKa cells). HEKa cells were cultured with heat-killed *C. acnes* (500 μg ml^−1^) and various concentrations of antimicrobial agents (ozenoxacin, nadifloxacin, and clindamycin) for 24 h. Culture supernatants were collected, and the concentrations of IL-6 and IL-8 were measured. The bars and the error bars represent the means and the standard errors for three independent experiments, respectively. *P < 0.05 compared with nontreated group (Dunnett’s multiple comparison test)

Ozenoxacin at the concentrations from 1 to 30 μg ml^−1^ significantly inhibited *C. acnes*-induced production of IL-6 and IL-8 in a dose-dependent manner (Fig. 1). The maximum inhibition rate of ozenoxacin was 64% for IL-6 and 67% for IL-8 at the concentration of 30 μg ml^−1^. Nadifloxacin also showed the significant inhibitory effects, at concentrations of 10 μg ml^−1^ and above, respectively (Fig. 1). The maximum inhibition rate of nadifloxacin was 50% for IL-6 and 45% for IL-8 at the concentration of 30 μg ml^−1^. In contrast to ozenoxacin and nadifloxacin, clindamycin showed no inhibitory effects on the production of these cytokines by HEKa cells (Fig. 1). Antimicrobial agents used in this study did not decrease viability of HEKa cells after incubation for 24 h up to the concentration of 30 μg ml^−1^ (an inhibition rate against cell viability was within 10%) (Supplementary Fig. 1).

### Effects of ozenoxacin on heat-killed *C. acnes*-induced pro-inflammatory cytokine production in human monocytes

The production of IL-1β, IL-6, IL-8, and TNF-α in THP-1 cells was increased after exposure to heat-killed *C. acnes* concentration dependently (from 0.5 to 500 μg ml^−1^, data not shown). The effects of antimicrobial agents on inflammatory cytokine production in THP-1 cells were evaluated after exposure to 50 μg of the bacteria per ml (~1 × 10^8^ CFU ml^−1^).

**Fig. 2:**
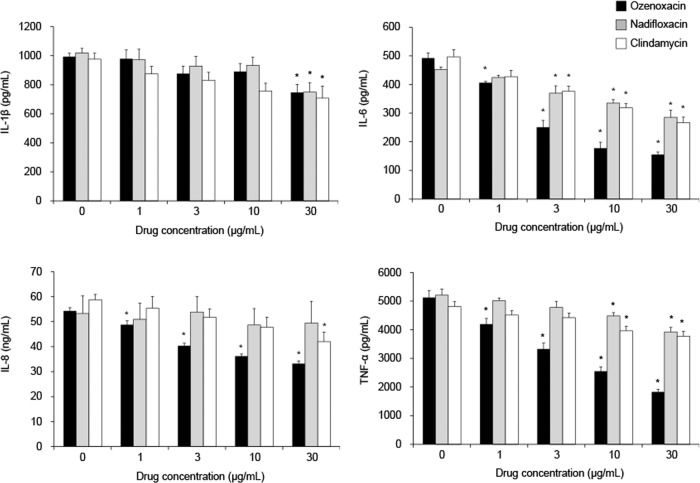
Effects of antimicrobial agents on heat-killed *C*. *acnes*-induced cytokine production in human monocytes (THP-1 cells). THP-1 cells were cultured with heat-killed *C. acnes* (50 μg ml^−1^) and various concentrations of antimicrobial agents for 24 h. Culture supernatants were collected, and the concentrations of IL-1β, IL-6, IL-8, and TNF-α were measured. The bars and the error bars represent the means and the standard errors for three independent experiments, respectively. *P < 0.05 compared with nontreated group (Dunnett’s multiple comparison test)

Ozenoxacin at concentrations from 1 to 30 μg ml^−1^ significantly inhibited the production of IL-6, IL-8, and TNF-α from THP-1 in a dose-dependent manner. As for the production of IL-1β, ozenoxacin at the concentration of 30 μg ml^−1^ showed significant inhibitory effects (Fig. 2). The maximum inhibition rate of ozenoxacin was 68% for IL-6, 39% for IL-8, 64% for TNF-α, and 22% for IL-1β at the concentration of 30 μg ml^−1^. Nadifloxacin and clindamycin also significantly inhibited IL-6 production at concentrations of 3 μg ml^−1^ and above (Fig. 2). The maximum inhibition rate of both nadifloxacin and clindamycin was 37 or 46% at the concentration of 30 μg ml^−1^. As for the other cytokines, clindamycin weakly reduced the production of IL-1β, IL-8, and TNF-α at the concentration of 10 or 30 μg ml^−1^ (Fig. 2). Nadifloxacin also weakly reduced the production of IL-1β and TNF-α at the concentration of 10 or 30 μg ml^−1^, and did not show clear inhibitory effect on the production of IL-8 (Fig. 2). Antimicrobial agents used in this study also did not decrease viability of THP-1 cells up to the concentration of 30 μg ml^−1^ (an inhibition rate against cell viability was within 10%) (Supplementary Fig. 2).

### Effects of ozenoxacin on heat-killed *C. acnes*-induced MAPK and NF-κB signaling in human keratinocytes and monocytes

MAPK signaling and NF-κB signaling are important for the regulation of the inflammatory response in keratinocytes and immune cells. To investigate whether these signaling pathways are involved in the anti-inflammatory activity of ozenoxacin and nadifloxacin, we used western blotting to examine the expression of related proteins in HEKa cells and THP-1 cells.

**Fig. 3:**
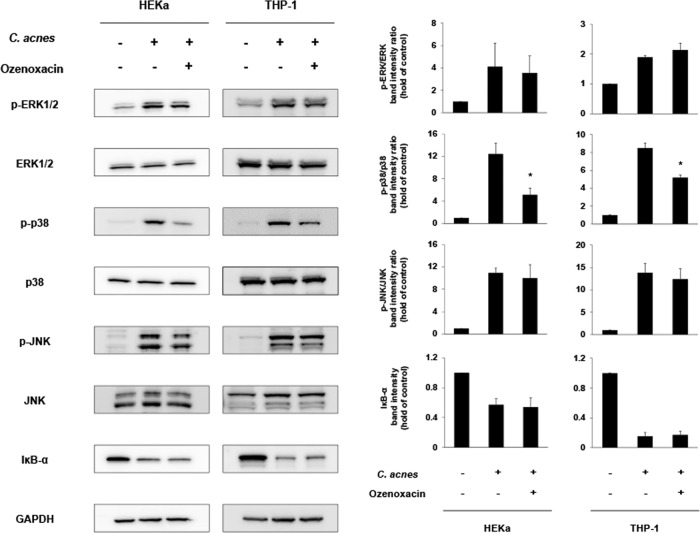
Effects of ozenoxacin on heat-killed *C*. *acnes*-induced MAPK and NF-κB signaling in human keratinocytes and monocytes. HEKa cells and THP-1 cells were incubated with heat-killed *C. acnes* (500 μg ml^−1^ for HEKa and 50 μg ml^−1^ for THP-1) and 30 μg ml^−1^ of ozenoxacin for 20 or 60 min. The expressions of p-ERK1/2, ERK1/2, p-p38, p38, p-JNK, JNK, and IκB-α were measured by western blot analysis. Band signal intensity was measured with image analysis software and normalized to GAPDH band intensity. As for ERK1/2, p38, and JNK, the relative level of protein phosphorylation was calculated as the ratio of phosphorylated to total protein. The bars and the error bars represent the means of hold of control group (no stimulation and no antimicrobials) and the standard errors for three independent sample, respectively. *P < 0.05 compared with control group (Student’s *t* test)

The phosphorylation of p38, JNK, and ERK1/2 was increased in HEKa cells and THP-1 cells after exposure to heat-killed *C. acnes* for 20 or 60 min. Moreover, the expression of IκB-α, an inhibitory factor of NF-κB nuclear translocation in both cells, was decreased after exposure to *C. acnes* (Fig. 3).

Ozenoxacin at the concentration of 30 μg ml^−1^ significantly inhibited the phosphorylation of p38 but had little effect on the phosphorylation of ERK1/2 and JNK and the degradation of IκB-α in both HEKa cells and THP-1 cells.

Nadifloxacin showed the inhibitory profiles similar to ozenoxacin which significantly inhibited only p38 phosphorylation. However, the inhibitory effects of nadifloxacin in THP-1 cells were not significant. In addition, nadifloxacin did not show the inhibitory effects in HEKa cells so much as ozenoxacin (inhibition rate of ozenoxacin was 59%, while that of nadifloxacin was 35%) (Supplementary Fig. 3).

### Effects of ozenoxacin on a rat model of heat-killed *C. acnes*-induced dermatitis

**Fig. 4:**
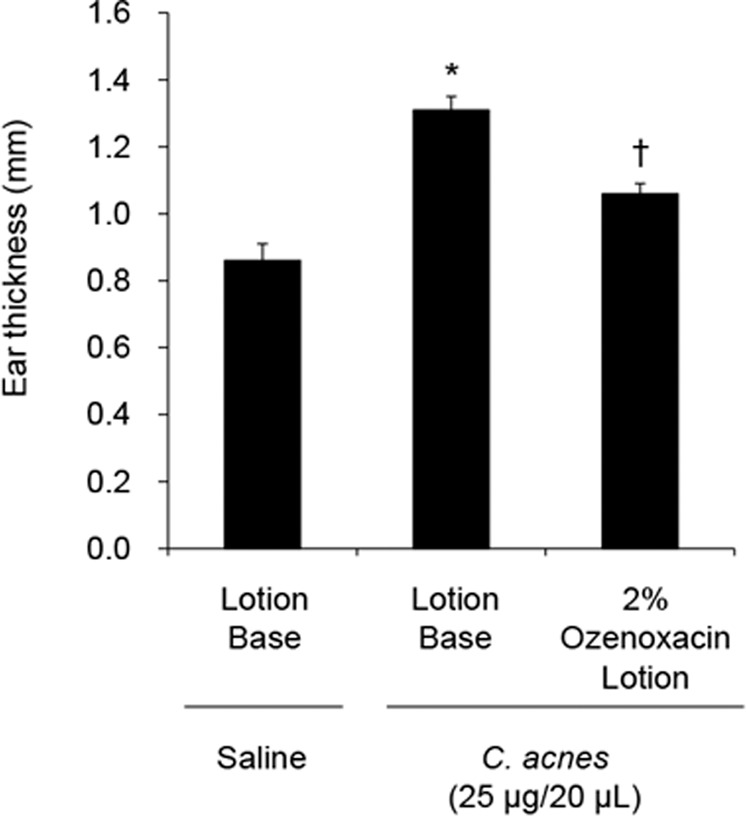
Effect of ozenoxacin on *C*. *acnes*-induced dermatitis in rat. Heat-killed *C. acnes* was injected intradermally into rat ear. Just before injection of the bacteria, the lotion containing 2% ozenoxacin, or the lotion base, was topically applied to each rat ear. Ear thickness was measured using a digital thickness gauge at 2 h after *C. acnes* injection. Reproducibility was confirmed by performing two independent experiments. The bars and the error bars represent the means and the standard errors for 11–14 animals, respectively. *P < 0.05 compared with group injected with saline and topically treated with the lotion base. ^†^*P* < 0.05 compared with group injected with *C. acnes* and topically treated with the lotion base (Student’s or Welch’s *t*-test)

On the basis of the in vitro results, we investigated the effects of ozenoxacin on the rat model of *C. acnes-*induced inflammation. Ear swelling with cutaneous erythema developed rapidly after intradermal injection of heat-killed *C. acnes* into rat ear. Just before the bacterial injection, the lotion containing 2% ozenoxacin or the lotion base was administered topically to rat ear. At 2 h after the administration, the lotion containing 2% ozenoxacin significantly reduced an increase in the ear thickness compared with the lotion base (Fig. 4).

## Discussion

In the present study, ozenoxacin inhibited the production of pro-inflammatory cytokines IL-6 and IL-8 in human epidermal keratinocytes, and IL-1β, IL-6, IL-8, and TNF-α by THP-1 cells stimulated by heat-killed *C. acnes* (Figs. 1 and 2). The cytokines inhibited by ozenoxacin are well-known initiators and promoters of inflammatory acne [2, 3]. IL-8 is a member of the CXC chemokine family and promoter of neutrophil migration to the pilosebaceous follicles. Release of lysosomal enzymes from these neutrophils leads to rupture of the follicular epithelium and further inflammation. IL-1β, IL-6, and TNF-α are multifunctional cytokines that can induce a broad range of secondary inflammatory effects in response to microbial infections. The expression levels of these cytokines are found to be upregulated in involved skin in acne patients [5, 11–13].

Topical antimicrobial agents, such as nadifloxacin and clindamycin, are widely used for the treatment of inflammatory acne, and these antimicrobial agents have been reported to have anti-inflammatory activities [17, 27–29]. Kuwahara et al. reported that nadifloxacin and clindamycin inhibited the production of IL-1β from PBMC stimulated by heat-killed *C. acnes* [17]. In addition, they also showed that nadifloxacin, not clindamycin, inhibited the production of IL-1α, IL-6, and IL-8 from keratinocytes stimulated by IL-1β and IFN-γ [17]. Consistent with those results, we observed that nadifloxacin, not clindamycin, inhibited the production of pro-inflammatory cytokines from keratinocytes (Fig. 1). On the other hand, clindamycin as well as nadifloxacin showed inhibitory effects on the production of cytokines from THP-1 cells (Fig. 2). Furthermore, we observed that ozenoxacin tended to inhibit the cytokine production from both of keratinocyte and THP-1 more strongly than nadifloxacin and clindamycin (Figs. 1 and 2). These results may suggest that ozenoxacin has a more potent anti-inflammatory activity against inflammatory acne than these other antimicrobial agents.

The exact molecular mechanism by which *C. acnes* activates the production of cytokines from keratinocytes or immune cells remains unknown, although many studies have shown pattern recognition receptors, such as TLRs, are involved [5, 6]. As for the inflammation induced by Gram-positive bacteria, including *C. acnes*, the activation of TLR2 by peptidoglycan, a component of the bacterial cell wall, is especially important [3, 9]. Some reports showed that TLR2 is involved in the production of pro-inflammatory cytokines by stimulation with *C. acnes* in monocytes and keratinocytes [5, 6]. When a microbial ligand, including peptidoglycan, binds to TLRs, intracellular signaling pathways, such as MAPK and NF-κB pathways, are activated, which lead to the transcription of cytokines via transcriptional factors, including activating protein-1 and NF-κB [4]. Indeed, previous studies have reported that exposure of keratinocytes and monocytes to *C. acnes* leads to the activation of the MAPK and NF-κB pathways in these cells [30, 31]. In addition, the related transcriptional factors are shown to be activated with consequent elevated expression of their target genes in inflammatory acne lesions [11]. As in previous studies, we observed the phosphorylation of MAPKs (ERK1/2, p38, and JNK) and degradation of IκB-α, an inhibitory factor of NF-κB, in keratinocytes and THP-1 cells stimulated by heat-killed *C. acnes* (Fig. 3). Regarding these intracellular pathways activated by *C. acnes*, the p38 phosphorylation pathway was markedly inhibited by ozenoxacin in both keratinocytes and THP-1 cells (Fig. 3). In addition, nadifloxacin, other topical quinolone antimicrobial agent, showed similar effects especially in keratinocytes (Supplementary Fig. 3). Li et al. have shown that p38 inhibitors with no effect on the phosphorylation of ERK1/2 or JNK inhibit the production of IL-1α, IL-8, and TNF-α in keratinocytes stimulated by live *C. acnes* [32]. From our results and those of Li et al., the phosphorylation of p38 may play an important role for induction of cytokine production in keratinocytes and THP-1 cells, and the inhibition of p38 phosphorylation by quinolones investigated in this study is considered to be one of the mechanisms underlying reduction in cytokine production. In addition, ozenoxacin tended to inhibit p38 phosphorylation induced by *C. acnes* more than nadifloxacin (Fig. 3 and Supplementary Fig. 3). Some differences between the inhibitory effects of ozenoxacin and those of nadifloxacin on cytokine production in *C. acnes*-stimulated cells may be partially related to the between-drug difference in inhibitory effects on p38 phosphorylation. Moreover, Li et al. also showed that p38 phosphorylation is upregulated in inflammatory acne [32]. Observations by Li et al. and us suggest that the inhibition of p38 phosphorylation by quinolones, such as ozenoxacin, leads to the exertion of its anti-inflammatory effects on acne patients.

The concentrations of ozenoxacin employed in the present study ranged from 1 to 30 μg ml^−1^, far higher than those needed for antibacterial activity against *C. acnes*. However, it was reported that the mean drug concentration in the pustules of acne patients after a daily application of the lotion containing 2% ozenoxacin for 3 days was 53.6 μg ml^−1^ [33]. The concentrations of ozenoxacin used in this study may be sufficient in the lesional skin of acne patients. In addition, the anti-inflammatory effects of the lotion containing 2% ozenoxacin were assessed in a rat model of acute dermatitis induced by *C. acnes*. In this study, heat-killed *C. acnes* was injected intradermally into the ear skin of rats to induce swelling. This animal model has been used to mimic the inflammatory response occurring in human acne upon follicular rupture [12, 25, 26]. The lotion containing 2% ozenoxacin inhibited ear swelling significantly compared with the lotion base at 2 h after *C. acnes* injection (Fig. 4).

In conclusion, in vitro and in vivo anti-inflammatory effects of ozenoxacin were observed in the present study. The anti-inflammatory effects of ozenoxacin are considered to contribute to the therapeutic efficacy for inflammatory acne.

## Supplementary information

Supplementary material
