# Supplementary material for: Anti-inflammatory effects of ozenoxacin, a topical quinolone antimicrobial agent
Source: J Antibiot (Tokyo). 2020 Jan 23;73(4):247–54. doi: 10.1038/s41429-020-0278-5 (PMC7056637; doi:10.1038/s41429-020-0278-5)
Supplement: Supplementary file 1 — Supplementary material [file 41429_2020_278_MOESM1_ESM.docx]

Ozenoxacin

Nadifloxacin

Clindamycin

**Supplemental Figure 1**

Cytotoxicity of each antimicrobial agent in HEKa cells was assessed by WST assay. HEKa cells were cultured with heat-killed *C. acnes* (500 μg/mL) and various concentrations of antimicrobial agents (ozenoxacin, nadifloxacin, and clindamycin) for 24 h. Thereafter, cell viability was measured by WST-1 reagent. The bars and the error bars represent the means and the standard errors for three independent experiments, respectively.

Ozenoxacin

Nadifloxacin

Clindamycin

**Supplemental Figure 2**

Cytotoxicity of each antimicrobial agent for THP-1 cells was assessed by WST assay. THP-1 cells were cultured with heat-killed *C. acnes* (50 μg/mL) and various concentrations of antimicrobial agents (ozenoxacin, nadifloxacin, and clindamycin) for 24 h. Thereafter, cell viability was measured by WST-1 reagent. The bars and the error bars represent the means and the standard errors for three independent experiments, respectively.

**Nadifloxacin**

**p-ERK1/2**

**ERK1/2**

**p-p38**

**p38**

**p-JNK**

**JNK**

**-**

**HEKa**

**-**


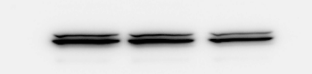

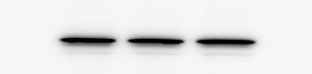

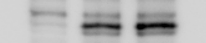

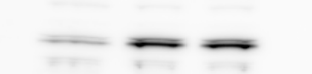

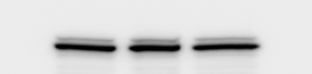

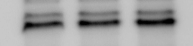

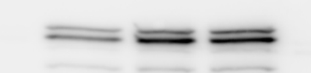

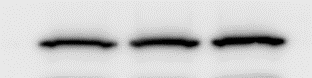

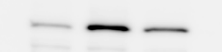


***C. acnes***

**+**

**+**

**-**

**-**

**THP-1**

**+**

**+**

**+**

**-**

**-**

**+**


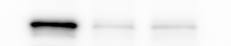

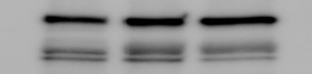

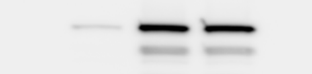

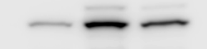


**IκB-α**


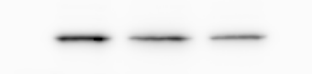

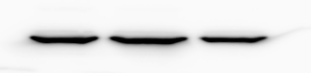


**GAPDH**


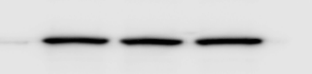

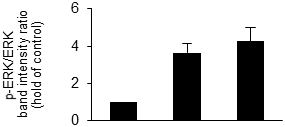

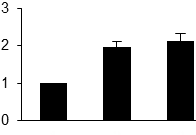

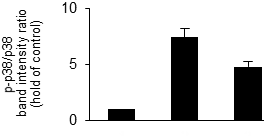

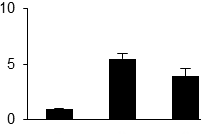

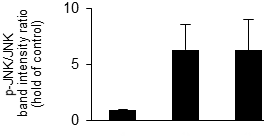

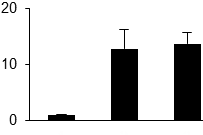

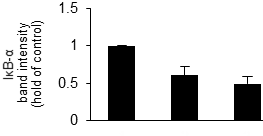

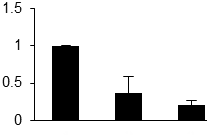


-

+

+

-

+

+

-

-

+

-

-

+

Nadifloxacin

*C. acnes*

HEKa

THP-1

*

**Supplemental Figure 3**

Effects of nadifloxacin on heat-killed *C. acnes*-induced MAPKs and NF-κB signaling in human keratinocytes and monocytes. HEKa cells and THP-1 cells were incubated with heat-killed *C. acnes* (500 μg/mL for HEKa, 50 μg/mL for THP-1) and 30 μg/mL of nadifloxacin for 20 or 60 min. Expressions of p-ERK1/2, ERK1/2, p-p38, p38, p-JNK, JNK, and IκB-α were measured by western blot analysis. Band signal intensity was measured with image analysis software and normalized to the GAPDH band intensity. As for ERK1/2, p38, and JNK, the relative level of protein phosphorylation was calculated as the ratio of phosphorylated protein to total protein. The bars and the error bars represent the means of hold of control group (no stimulation and no antimicrobials) and the standard errors for three independent samples, respectively. *P < 0.05 compared to control group (Student’s *t*-test).
